# Understanding and measuring mechanical signals in the tumor stroma

**DOI:** 10.1002/2211-5463.13923

**Published:** 2024-11-10

**Authors:** Fàtima de la Jara Ortiz, Chiara Cimmino, Maurizio Ventre, Alessandra Cambi

**Affiliations:** ^1^ Department of Medical BioSciences Radboud University Medical Center Nijmegen The Netherlands; ^2^ Department of Chemical, Materials and Industrial Production Engineering University of Naples Federico II Naples Italy; ^3^ Center for Advanced Biomaterials for Healthcare@CRIB Fondazione Istituto Italiano di Tecnologia Naples Italy; ^4^ Interdisciplinary Research Centre on Biomaterials University of Naples Federico II Naples Italy

**Keywords:** cancer‐associated fibroblast, extracellular matrix, mechanobiology, stiffness, tumor mechanics

## Abstract

The tumor microenvironment (TME) is well known for its immune suppressive role, especially in solid tumors which are characterized by a thick, dense stroma. Apart from cell–cell interactions and biochemical signals, the tumor stroma is also characterized by its distinct mechanical properties, which are dictated by the composition and architecture of its extracellular matrix (ECM). Cancer‐associated fibroblasts (CAFs) are the main producers and remodelers of the stromal ECM, and their heterogeneity has recently become a focus of intense research. This review describes recent findings highlighting CAF subtypes and their specific functions, as well as the development of 3D models to study tumor stroma mechanics *in vitro*. Finally, we discuss the quantitative techniques used to measure tissue mechanical properties at different scales. Given the diagnostic and prognostic value of stroma stiffness and composition, and the recent development of anti‐tumor therapeutic strategies targeting the stroma, understanding and measuring tumor stroma mechanical properties has never been more timely or relevant.

## Graphical abstract

This review discusses cancer‐associated fibroblast subtypes and their functions, particularly in relation to extracellular matrix production, as well as the development of 3D models to study tumor stroma mechanics *in vitro*. Several quantitative techniques to measure tissue mechanical properties are also described, to emphasize the diagnostic and prognostic value, as well as the therapeutic relevance of stroma stiffness and composition.

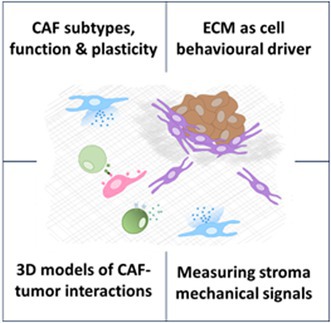

**Abbreviations**
AFM: atomic force microscopy
BM: Brillouin microscopy
CAF: cancer‐associated fibroblast
ECM: extracellular matrix
LOX: lysyl oxidase
MDSCs: myeloid‐derived suppressor cells
MPT: multiple particle tracking
MR: magnetic resonance
MSC: mesenchymal stem cell
OC: Optical Coherence
PA: Photoacoustic
RT‐DC: real‐time deformability cytometry
SW: shear wave
TAGLN: transgelin
TFM: traction force microscopy
TME: tumor microenvironment
US: ultrasound
αSMA: α‐smooth muscle actin

In many solid tumors, the presence of a poorly vascularized, dense and extensive stromal compartment in the so‐called tumor microenvironment (TME) correlates with tumor progression and bad prognosis. The TME includes different types of cells (e.g.: fibroblasts, macrophages, dendritic cells, T cells etc), proteins (e.g.: collagen, laminin, fibronectin etc) and other biomolecules (e.g.: prostaglandins, cytokines and growth factors) around and within the tumor mass, which are collectively known as stroma. The intricate interplay among all these cellular and molecular components dictates whether the TME if pro‐ or anti‐tumorigenic. Therefore, the TME is associated with tumor invasiveness, decreased drug efficacy and therapy resistance which ultimately results in poor prognosis [1, 2, 3].

In the TME, fibroblasts can differentiate into cancer‐associated fibroblasts (CAFs), a highly heterogeneous cell population that directly affects tumor growth and invasion, and significantly impact therapeutic outcomes [4]. Known for being the major stroma organizers, CAFs produce and remodel the extracellular matrix (ECM) and secrete cytokine and growth factors that modulate the tumor as well as the stromal immune cells [4, 5, 6, 7] (Fig. 1A). Numerous studies have demonstrated that the ECM biophysical properties such as stiffness or alignment play important roles in controlling cancer cell proliferation, differentiation and invasion [2, 8, 9, 10, 11]. It is therefore not surprising that in several solid tumors, aberrant ECM composition and organization are strongly associated with therapy resistance and poor prognosis [12, 13].

**Fig. 1:**
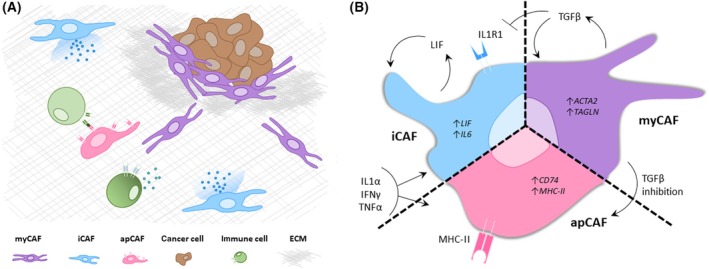
CAF subtypes shape the TME. (A) The TME and its constituents. The TME includes the tumor lesion as well as the surrounding stroma. In the TME, the stromal ECM can become thicker and more aligned and this is known to facilitate tumor growth and invasion. myCAFs are the major ECM producers while iCAFs secrete cytokines and modulate the immune environment. (B) Phenotypic plasticity of CAFs. CAFs can undergo phenotypic changes as a consequence of converging stimulatory pathways. TGFβ is known as the main inducer of myCAF phenotype. Inhibition of TGFβ has been reported to promote phenotypic changes towards apCAF phenotype. In addition, TGFβ is suggested to inhibit IL1R1 production in iCAFs. Inflammatory pathways can lead to iCAF and apCAF induction; however, the exact signaling pathway still needs to be elucidated.

With respect to the surrounding healthy tissue, the ECM composition and architecture of the tumor stroma confer characteristic mechanical properties to the TME (e.g. increased stiffness), which may have both a diagnostic and a prognostic value [14]. As more insights into tumor stroma mechanics become available, CAFs and stromal ECM are increasingly seen as interesting targets for novel anti‐cancer therapeutical approaches [15, 16, 17]. This review aims to highlight differences and similarities among the different CAF subtypes and to briefly describe the *in vitro* preclinical models developed to mimic the cellular and non‐cellular complexity of the TME. Furthermore, the importance of the ECM biophysical properties in shaping the TME is discussed. Finally, we will describe novel quantitative approaches currently developed to measure pathological changes in tissue stiffness, which will likely offer additional parameters for diagnostic and/or prognostic purposes.

## Cancer‐associated fibroblasts; subtypes, functions, and plasticity

Cancer‐associated fibroblasts (CAFs) are main producers and crucial regulators of the stromal compartment within the tumor microenvironment (TME), where they can greatly influence tumor growth and dissemination [18]. Single cell RNA sequencing (scRNAseq) analysis of the TME from several cancer tissue types has revealed that CAFs are a highly heterogeneous populations, characterized by distinct sets of expressed genes, cellular ontogeny and also location with respect to the tumor [19, 20, 21] (Fig. 1B). Significant efforts have therefore been devoted to classify CAFs into different subtypes to comprehend their contribution to the TME. This classification can be of phenotypic or functional nature, oftentimes resulting in one single CAF being grouped simultaneously into different subtypes. As fibroblasts can phenotypically change in response to a specific stimulus, it is not only complex to establish a robust CAF subtype classification, but also very challenging to generate accurate models to study this particular cell type [20, 21, 22, 23].

Despite CAFs' ability of undergoing different cellular states, common ground has been found when defining function‐based CAF subtypes. Certain specific subtypes have been repeatedly described in different studies, providing CAF subtype standardization: these subtypes are known as myofibroblast CAFs (myCAFs), immunomodulatory CAFs (iCAFs) and antigen‐presenting CAFs (apCAFs) [20, 24, 25, 26, 27, 28] (Fig. 1B). The myCAFs have been defined as α‐smooth muscle actin (αSMA)‐and transgelin (TAGLN)‐high expressing CAFs with a contractile phenotype, located in close contact with cancer cells [20]. They are shown to be TGFβ‐inducible and are generally described to produce and remodel the ECM [22]. The iCAFs are reported to be IL1‐inducible, and present a secretory phenotype supported by high expression of the pro‐inflammatory cytokines IL6 and LIF [24, 29]. They are mostly located in the surroundings of cancer cells, however not in direct contact [20]. The apCAFs express high levels of CD74 and MHC‐II and are suggested to be regulated by IFNγ *in vivo* [26]. Interestingly, conflicting reports indicate apCAFs can be both pro‐ and anti‐tumorigenic [26, 30, 31, 32].

A detailed overview of CAF subtypes and the specific markers used for their classification falls outside the scope of this review, and for this, we redirect the reader to several recent publications [4, 17, 19, 22, 23, 33]. Here, however, we find it important to highlight that depending on its anatomical location, the same CAF subtype can slightly differ among different tissues and organs. This heterogeneity in the expression levels of CAF subtype‐specific markers is evident when publicly available databases of scRNAseq of different tumor tissues are compared. For example, in the myCAFs from pancreatic ductal adenocarcinoma (PDAC) the levels of the myCAF marker Thy1 are clearly much higher than in iCAFs and apCAFs [26], but in breast cancer, no significant differences in Thy1 levels are found among the different subtypes [27, 34]. Furthermore, in breast cancer iCAFs, no expression of the typical myCAF marker Tagln has been reported [27], whereas PDAC iCAFs do express Tagln at the same or slightly lower levels as compared to myCAFs [26, 35]. Analogously, the expression of the classical iCAF marker IL6 has also been reported in myCAFs from melanoma, head and neck squamous cell carcinoma as well as lung cancer [36]. These examples indicate that CAF subtype‐specific markers exist and are useful to classify these cells, but their expression patterns might be less well‐defined than anticipated. Therefore, as recently proposed by Cords and colleagues, besides a marker‐based classification, a function‐ and location‐driven CAF classification system is more and more needed to better understand CAF subtypes and their exact functions within the TME [33].

Besides a different ontogeny, another aspect significantly contributes to CAF heterogeneity: some CAF subsets are not permanent but interconvertible via activation of specific signaling pathways [5, 37, 38, 39, 40]. Plasticity between myCAFs and iCAFs has been reported in different studies, apparently driven by the interplay between TGFβ and IL1α [20, 24]. Efforts have been made to elucidate the interaction between the activated pathways SMAD2/3 and JAK/STAT, suggesting that TGFβ has an inhibitory effect on the expression of the IL1α receptor [24]. Interestingly, inhibiting TGFβ does not promote the expected phenotypical change towards iCAF, but rather converts into another subtype, namely interferon‐licensed CAF, which shares characteristics with the previously described apCAF phenotype [27]. In addition, apCAFs have been shown to switch to myCAF‐specific gene expression when cultured in 2D (possibly due to autocrine TGFβ signals induced by the stiff culture substrate [41], highlighting their ability to adjust to the environment [26]).

Understanding the molecular mechanisms driving CAF subtype plasticity will be essential for the cancer biology research field and detailed studies of CAF ontogeny in the future are warranted.

## CAF‐derived ECM as a behavioral driver of cancer and stromal cells

One of the most impactful functions of CAFs within the TME is production, deposition, and remodeling of the ECM, and this function has predominantly been assigned to myCAFs [17]. However, new insights provided by recent single cell transcriptomics suggest that iCAFs and other CAF subtypes might contribute as well [26, 42, 43]. Different CAF subtypes may supply different ECM components in a distinct manner [44], and more insight into the specifics of their contribution might provide new leads for specific targeting approaches. Besides synthesizing the ECM, CAFs are also known to exert intense contractile forces on the ECM, thereby making it denser and stiffer [45]. This process is part of the so‐called desmoplastic reaction, which also leads to blood vessel formation and immune cell infiltration. Certain histological features of desmoplasia correlate with tumor aggressiveness, hence the importance of understanding the molecular and mechanical determinants of this reaction. In particular, CAFs contractile behavior is affected by local anisotropy. CAFs cultured on synthetic aligned fibrous matrices exhibit a myofibroblastic phenotype and promote features similar to desmoplasia, achieved through the formation of force‐generating lateral protrusions [46]. Moreover, CAFs contribute to stromal stiffening through collagen crosslinking as they express several extracellular enzymes of the lysyl oxidase (LOX) family [45]. This tumor stroma matrix shows biophysical and biochemical characteristics which are distant from healthy tissues and can in turn regulate CAF functions and fate as well. Indeed, stromal stiffness is critical for inducing and maintaining CAF phenotype and contributes to mesenchymal stem cell (MSC) differentiation into CAFs [47, 48]. Hence, a positive feedback loop exists between CAFs and the aberrant stromal matrix they produce, which reinforces the pro‐malignant nature of the stromal microenvironment.

The aberrant biochemical and biophysical properties of the stromal ECM have also repercussions on the behavior of other TME resident cells, starting with cancer cells [16]. For example, the ECM represents an alternative source of nutrients under amino acid starvation and supports cancer cell growth through tyrosine catabolism [49]. The ECM can also promote tumor budding by fibronectin‐sustained intercellular force transmission between CAFs surrounding tumor clusters [50]. In addition, fiber alignment of hyaluronan and proteoglycan link protein 1 by CAFs promotes tumor cell invasion and migration [51]. Lastly, collagen I knock‐out in myCAFs resulted in an altered chemokine profile by cancer cells, which led to recruitment of myeloid‐derived suppressor cells (MDSCs) and defective recruitment and activation of B and T lymphocytes [52].

Stromal ECM architecture and composition can directly affect immune cells as well [53]. T cell motility *in vitro* is modulated by the alignment and density of collagen fibers, and T cells increase their speed in aligned matrices, where they move along the axis of the fibers [54]. On the other hand, they are observed to reduce their motility in higher collagen concentrations, which is consistent with *in vivo* findings [55, 56]. T cells encapsulated in high‐density matrices display a weakened ability to eliminate tumor cells, supported by whole transcriptomics analysis showing an increase of regulatory T cell markers and downregulation of cytotoxic T cell markers [55]. This event can be explained by CD4+ T cell cytoskeleton modulation through ECM stiffness sensing, resulting in activation suppression [57]. Innate immune cells, such as macrophages, can also respond to the ECM. For instance, matrix stiffness promotes upregulation of the YAP/TAZ pathway, which stimulates DAB expression in macrophages and leads to ECM remodeling and integrin‐dependent migration [58]. In fact, decellularized tumor tissue repopulated by naïve healthy macrophages has been shown to educate them into tumor‐associated macrophages (TAMs), as they share their genetic profile with TAMs found in the tumor tissue [59].

As several studies indeed confirm the role of the ECM in disease development, targeting of the CAF‐ECM axis is emerging as an appealing approach to modulate ECM properties and facilitate cancer eradication. For example, targeting of LOX resulted in less stiff matrices and increased intratumoral T cell infiltration [60]. In addition, specific depletion of LRRC15+ myCAFs *in vivo* led to reduced fibroblast and tumor content, CAF‐to‐fibroblast polarization and increased CD8+ T cell activity [61]. Also, inhibition of intracellular collagen trafficking in dermal fibroblasts by a newly developed peptide inhibitor targeting TANGO1 and cTAGE5 interaction led to a reduced ECM component secretion in fibrotic processes [62]. Fibrotic tumors, such as CSM4 colorectal cancer and pancreatic ductal adenocarcinoma, could benefit from these fibrosis‐targeting therapeutic approaches.

## 3D models to study CAF‐tumor interactions

To date, studies on the TME mostly rely on cell cultures in 2D or on cell spheroids. These systems do not require specialized equipment, are readily accessible for inspections and rely on well‐established methodologies. Yet, cell monolayers and spheroids do not fully capture the heterogeneous composition and structural complexity of the TME as they lack the stromal components. Attempts to reproduce this complexity are represented by cocultures, either on flat surfaces or in spheroids, of different cell types including stromal cells, immune cells or endothelial cells [63, 64, 65]. Here, although a certain level of cell self‐organization is visible, a tight control of cell positioning like in real tumor tissues cannot be achieved. Recent advancements in 3D printing and bioprinting technologies enable to fabricate cellular constructs that better recapitulate TME both in terms of structure and composition. For instance, a 3D system of triple‐negative breast cancer stem cells with a stromal compartment supported by decellularized breast ECM bioink has been recently proposed as a platform for drug discovery [66].

A central feature of tumors is their heterogeneity that cannot be reproduced *in vitro* with cell lines. In the last two decades, organoids have emerged as a ground‐breaking technology to regenerate complex tissues *in vitro* by cultivating different cell types, including iPSC, stem cells or tissue‐derived cells, in well‐defined physical–chemical conditions [67]. In addition, organoids derived from primary tumor cells have been shown to reproduce histological features observed in tumors *in vivo* [68, 69]. To date, numerous protocols have been developed for the production of libraries of tumor organoids that are able to recapitulate the spatiotemporal tumor heterogeneities. Cocultures with other cell types, including fibroblasts, has enriched the structural complexity of the organoids [70, 71, 72]. Despite these benefits, the organoid technology still presents some limitations: the *in vitro* generation of 3D tumors may require weeks or months, which limits high throughput studies; no standardized protocols exist for cell manipulation and culture; the yield is inconsistent [73].

In the last few years, microfluidics devices have become increasingly useful to better control the TME conditions *in vitro*. Networks of microfluidic channels can generate gradients of soluble molecules, fluid pressure or shearing fluid forces. Several studies report the use of microfluidic systems in combination with cancer cells alone or cocultured with CAFs [74, 75], lymphatic endothelial cells [76] or vascular endothelial cells [77, 78], and even tumor microtissues [79]. This has paved the way for the development of tumor‐on‐chip devices, i.e. systems allowing to control and monitor the biochemical and biophysical characteristic of miniaturized TMEs [80]. With the use of arrays of chambers and channels, enabling to precisely localize and connect cell clusters, complex phenomena occurring in tumor progression including neoplastic cell migration and stromal remodeling can also be investigated in a systematic and controlled manner [79]. Very recently, a microfluidic system of colorectal cancer cells revealed a decreased sensitivity of these cells to the EGFR inhibitor gefitinib when they were cocultured with CAFs [81], highlighting the versatility of these devices.

Despite these interesting developments, it remains challenging to study the interplay between CAFs (and other cell types) and the stromal ECM, as spheroids and organoids require the use of culturing “scaffolds” such as fibrillar collagen gels or ECM extracts (e.g. Matrigel) that do not faithfully reproduce the complexity of the TME stromal ECM. Furthermore, complex 3D scaffold makes it difficult visualize cells with high spatial resolution or to have “access” to cells for direct stimulation, for instance mechanical or chemical conditioning can be done macroscopically, whereas local stimulation requires specialized equipment. Interestingly, cells on 1D structures display morphological and migratory features typical of 3D settings, but not observed in 2D [82, 83]. Using 1D electro spun polycaprolactone mats, Jiao *et al*., [84] showed that myofibroblast differentiation is affected by the spatial fiber arrangement as it is improved on randomly oriented mats as compared to aligned ones. As micro‐ and nanofabrication technologies are rapidly and steadily evolving, the integration of these technologies with rapid prototyping methods and with the aid of new materials will enable the fabrication of TME mimics capable of replicating specific chemical–physical characteristics of the pathological environment.

## How to measure mechanical signals of the stroma

An accurate assessment of CAF‐generated forces and stroma mechanical properties could provide relevant information for a better identification of the stages of cancer progression. Several investigative techniques can be used to measure various aspects of CAF or stroma mechanics, including pulling forces, pressure, elastic and viscoelastic properties, and their morphology (Table 1).

**Table 1:** Overview of quantitative techniques to measure mechanical properties of cells and tissues.

	Force/modulus range	Measurement	Spatial resolution	Advantages	Disadvantages
AFM	10 pN – 100 nN [111]	Elasticity, viscoelasticity, adhesion forces	1–10 nm [112]	High spatial resolution; high sensitivity; overlaps imaging and force maps; single molecule level	Complex and expensive; slow data acquisition; moduli are estimated from mathematical models.
TFM	1 pN – 100 nN [113]	Cell forces	100 nm [114]	Cell‐environment interaction; Non‐invasive	Requires cell/protein labeling; might be computationally intensive; limited to compliant substrates.
MPT	0.001 Pa – 10 kPa [115]	Particle displacements, viscoelasticity	10 nm	Local mechanical properties; highly sensitive; inexpensive	Requires microparticle labeling and injection in media/cells; relatively low throughput; difficult to perform in 3D media
RT‐FDC	0.1 kPa – 100 kPa [116]	Cell Deformation	300 nm [117]	Fast and non‐invasive; analyses of large cell populations; no cell labeling required	Limited spatial resolution; limited sensitivity to small differences in deformability; analysis of cell aggregates and tissues not straightforward
US	1 kPa – 100 kPa [118]	Elasticity	10 mm	Non‐invasive; inexpensive; portable; large field‐of‐view	Low spatial resolution; limited accuracy
MR	0.1 kPa – 10 kPa [119]	Elasticity	10 mm [119]	Non‐invasive; high contrast resolution for various tissues; large field‐of‐view	Expensive; requires specialized equipment
OC	1 kPa – 10 GPa [120]	Elasticity	1–10 μm	Label‐free measurement; high spatial resolution; real‐time imaging	Limited penetration depth: Expensive technique
PA	0.1 kPa – 100 kPa [121]	Elasticity, viscoelasticity	10 μm [121]	Provides both imaging and mechanical properties; micrometric resolution	Expensive; requires specialized equipment; limited depth penetration
BM	1–10 GPa [122]	Longitudinal modulus, viscoelasticity	1 μm [123]	Provides both imaging and mechanical properties; high resolution	Complex instrumentation; limited clinical applications; results are not directly related to cell/tissue elasticity; limited depth penetration

Atomic force microscopy (AFM) is probably one of the most popular techniques to study elastic and viscoelastic properties of both cells and tissues as well as adhesive forces between cells or macromolecules [85, 86]. By providing both images of surface topography and mechanical force measurements, AFM can correlate morphology and mechanics. The working principle is based on measuring the deflection of an elastic cantilever with a laser and then, using mechanical models, elastic or viscoelastic moduli can be estimated [87]. In the context of characterizing CAFs, stroma and their interactions, AFM has shown differences in the stiffness of normal fibroblasts and CAFs. For instance, normal pancreatic fibroblasts were found to be 1.3 times stiffer than pancreatic CAFs [88]. Also, in a rat mesentery mouse model of the basement membrane, colon cancer cells and CAFs extensively altered the microarchitecture and elasticity of the matrix making it softer and more inhomogeneous, traits that are more permissive to cancer cell invasion [89]. Recently, thanks to the AFM micron scale accuracy, stiffness measurements were combined with an artificial intelligence algorithm able to capture micrometric features of the collagen network (fiber linearity, thickness, cell proximity) of the tumor stroma to predict its elasticity [90]. The algorithm was able to link stoma mechanics with molecular indicators of EMT and tumor aggressivity (Twist1, ZEB1 and SLUG) in human breast cancer samples. This study emphasizes the use of artificial intelligence methods to develop efficient algorithms that integrate mechanics, ECM morphology and biomolecular data for diagnostic or prognostic purposes.

Traction force microscopy (TFM) measures cell generated forces by measuring the force‐induced displacement of fluorescent fiducial markers in a medium (compliant substrate or 3D gel) of known mechanical properties. The spatial force resolution depends on the ability to precisely measure the markers displacement by fluorescence microscopy and on the accuracy in the estimation of the material constants of the medium. TFM has been used to characterize the contractile behavior of CAFs and the dynamic interplay between CAF and colorectal cancer cells. CAFs exhibited a supracellular organization through fibronectin cables to build‐up compressive stresses within intratumoral capsules. The increased CAF‐induced compressive stresses promote cancer cell packing and a release of nuclear tension, which in turn favors YAP nuclear export [50]. Other force measurement techniques have been developed that share with TFM the principle of estimating the applied force from strain measurements. For example, micropost, or micropillar, arrays and nanonet force microscopy allow to measure the force exerted by cells in contact with these structures, posts or wires, by measuring their deflection [91, 92]. Differently from TFM, these techniques are based on regular arrays of structures, which simplifies the analysis and measurement methods while maintaining a high sensitivity at the expense of a non‐immediate fabrication of the substrates.

Similar to TFM, multiple particle tracking (MPT) rheology tracks thermal fluctuations of nanoparticles within gels, cells, or tissues. From the particle tracks, MPT rheology can deduce information on the viscoelastic properties of the medium. Although not routinely used in CAF‐stroma studies, MPT has been employed to study the different microrheological behavior of CAF or normal fibroblast (NF) spheroids of *in vitro* generated microtissues. While the microrheological behavior of CAFs or NFs does not change when they form spheroids, CAFs embedded in their own matrix have a more fluid‐like cytoskeleton as compared to the NFs, which might be a consequence of the different fibrillar nature of the synthesized matrices [93].

The techniques mentioned above suffer of a limited throughput allowing to analyze a few cells per hour. An emerging technique that dramatically increases the throughput (100–1000 cells/s) is the real‐time deformability cytometry (RT‐DC), a label‐free technology that measures cell deformation under hydrodynamic forces in microfluidic channels [94]. Using RT‐DC, Jaesche *et al*. reported an increased stiffness of CAFs isolated from malignant prostate cancer compared to non‐malignant prostatic fibroblasts (NPFs) [95]. Also, benign epithelial prostate cells cocultured with CAFs showed a decreased stiffness as compared to those cocultured with NPFs, suggesting the induction of an invasive phenotype [95].

These techniques are extremely useful to study the mechanical properties of cells and stroma that, together with the biochemical properties, could enhance the diagnostic or prognostic outcomes of tumor tissue analyses. While they can be used *in vitro* or in *ex vivo* (e.g. liquid biopsies) settings, they cannot be implemented in *in vivo* measurements. To obtain elastographic maps *in vivo*, other methods exist, some of which are routinely used in the clinic, while others are still at developmental or conceptual stage.

Ultrasound (US) elastography can assess variation in tissue stiffness, thus providing evidence of tumor onset or progression. Several US‐based elastographic methods have been developed so far. Among these, shear wave (SW) elastography provides quantitative elastographic maps by measuring the propagation speed of waves into the tissue. SWs are generated in the tissue with a focused beam of US waves, which can penetrate a few millimeters in the tissue. SW‐US elastography can inform about tumor stiffness and its changes along with changes in stroma components. Riegler *et al*. [96] found a significant correlation between the *in vivo* stiffness of syngeneic tumors (B16F10, EMT6, 4T1, KPR3070) measured with SW‐US and the stiffness measured on the tumors *ex vivo* through mechanical compression. Also, *in vivo* stiffness correlated with the total collagen concentration and immature collagen cross links of the tumor stroma, as well as with the density of activated fibroblasts.

Similar to SW‐US elastography, Magnetic Resonance (MR) elastography exploits the propagation of SWs to estimate the local tissue stiffness. In MR elastography, SWs are generated by an external electromechanical transducer on the surface of the body. Then, MR imaging is used to evaluate SW speed and wavelength. Stiffer tumor tissues enable waves to propagate faster hence emphasizing a mechanical contrast with the surrounding softer tissues. Using MR elastography Kader *et al*. [97] analyzed tissue samples from two prostate cancer xenograft mouse models, PC3 and LNCaP, and found increased prostate tumor stiffness and decreased water mobility in the former compared to the latter and the increase was associated with higher collagen and elastin levels.

US‐based techniques lack in spatial resolution, which is in the millimeter range. Techniques using different energy sources, e.g. light, provide much more detailed elasticity maps. For example, Optical Coherence (OC) elastography involves the application of static or dynamic mechanical stresses (like US and MR elastography) and measures tissue deformation using OC tomography that is an interferometric technique that generates images by measuring delays and intensities of waves back‐reflected from the tissue, with respect to a reference wave. Using light rather than US, OC elastography has a much higher spatial resolution (in the micrometer range), but its penetration depth is, however, still limited to a few millimeters [98]. Plekhanov *et al*. [99] used OC elastography for the morphological segmentation and mechanical characterization of an ectopic mammary carcinoma in mice. They were able to segment an area containing viable, dystrophic and necrotic tumor cells as well as edema, which highly correlated with histological results. Moreover, they retrieved detailed mechanical data showing that the heterogeneities in the tissue stiffness maps were related to the morphological alterations [99].

Photoacoustic (PA) elastography is another optical technique that is gaining popularity for its improved spatial resolution and versatility. The technique is based on the generation of mechanical (acoustic) waves by laser light pulses in the visible or near infrared region that propagate through a medium or tissue. As acoustic waves travel faster in stiffer regions, a map of stiffnesses can be generated. Despite its potentials, studies related to PA elastography in the assessment of the physical features of the tumor microenvironment (TME) are limited. Interestingly, a modified version of elastography accounting for tissue viscoelasticity was used for the characterization of an ectopic breast cancer in mice [100].

A very promising label‐free technique that can produce micron scale elastographic maps is the Brillouin microscopy (BM) [101]. It is based on the inelastic scattering of light with the thermally induced acoustic waves, known as phonons. The interaction causes a frequency shift in the scattered light that is related to the material stiffness. Maps of mechanical properties with a spatial resolution of ~1 μm can be acquired with this technique. BM has been extensively used to measure the mechanical properties of cells and biological tissues like cornea [102], skin [103], and cartilage [104]. In cancer research, Mahajan *et al*. [105] measured Brillouin shifts in spheroids of different cell types (MCF‐7, MDA‐MB‐231, PANC1 and PC3) cultured in various PEG‐heparin 3D gels. Spheroids adapted the microenvironment by changing their mechanical properties, i.e. spheroids growing in stiff degradable hydrogels are characterized by increased elastic moduli, as compared to spheroids in compliant degradable hydrogels. Also, spheroid mechanics and cell invasiveness are modulated by matrix stiffness as MDA‐MB‐231, PANC1 and PC3 spheroids formed in stiffer degradable hydrogels, showed higher elastic moduli and were less invasive compared to those in compliant degradable hydrogels [105]. However, the implementation of BM as cancer diagnostic tool is still far away, due to the relatively small penetration of the incident light, long acquisition times and non‐optimal radiation dosages.

The techniques described above provide a comprehensive set of tools which are effective at different length scales and can probe different stiffness ranges. Integrating high‐resolution imaging, detailed mechanical data and molecular analyses would certainly improve our understanding of the mechanobiology of CAFs and on the impact of tumor stroma on the evolution of the disease. This could ultimately contribute to develop novel diagnostic devices or possibly novel therapeutic agents targeting ECM compounds of the tumor stroma.

## Conclusions and open questions

The mechanical properties of the stroma as dictated by the dynamic interplay among CAFs (and CAF subtypes), their ECM, tumor‐resident immune cells and cancer cells are increasingly acknowledged as important orchestrators of tumor growth and invasion as well as novel diagnostic tools. It is therefore not surprising that significant efforts are invested in the development of *in vitro* models able of recapitulating the cellular and non‐cellular complexity of the tumor stroma. Very recently, human stem cell‐derived intestinal organoids have been described that were formed through self‐organization of epithelial organoids and autologous tissue‐resident memory T cells [106], thus paving the way to future studies of tissue‐resident immune responses in the TME. At the same time, the repertoire of quantitative techniques able to measure various aspects of cell and tissue mechanics at different scales is expanding. Single cell analysis has revolutionized the cancer research field, offering novel insights in the complexity and heterogeneity of the cell types populating and organizing the TME. However, these insights will have to be combined with knowledge about the ECM composition and architecture of the tumor stroma to obtain a more complete picture of the factors contributing to the immune suppressive TME of solid tumors. Combinations of anti‐tumor immunotherapy with targeting strategies aimed at modulating the TME immune suppression via controlled alteration of its mechanical properties might increase therapy efficacy. Recently, Rwandamuriye *et al*. [107] developed a biodegradable hyaluronic acid‐based hydrogel for sustained intraoperative delivery of Toll‐like receptor 3 agonist poly(I:C) that significantly reduced tumor recurrence after surgery in multiple mouse models. The poly(I:C) local release was found to reshape the TME by attracting inflammatory monocytes and depleting regulatory T cells. Similarly, biomaterials with mechano‐immunomodulatory properties could be used during surgery to repress CAF activity and prevent tumor recurrence. In the coming years, we envisage numerous studies focused on in‐depth preclinical and clinical testing of approaches aimed at understanding and modulating the TME mechanical properties: mechano‐immunology [108, 109] and mechano‐oncology [110] are coming of age.

## Conflict of interest

All authors do not have any actual or potential conflict of interest including any financial, personal or other relationships with other people or organizations within 3 years of beginning the submitted work that could inappropriately influence, or be perceived to influence, their work.

## Author contributions

AC and MV conceived the review. All authors contributed to manuscript writing and editing. AC supervised the project.
